# Catching Threads in Bacterial Cell Walls

**DOI:** 10.1021/acscentsci.2c01070

**Published:** 2022-10-11

**Authors:** Till Kallem, Lynette Cegelski

**Affiliations:** Department of Chemistry, Stanford University, Stanford, California 94305, United States

Urgent action is needed to stop the spread of bacterial infections. In 2019, 1.27 million lives were lost to antimicrobial resistant bacterial infections globally, and more than 100,000 were attributed to methicillin-resistant *Staphylococcus aureus*.^1^ In the U.S., *S. aureus* is a leading cause of healthcare-associated infections. The major burden of *S. aureus* is attributed to surgical site infections, including abdominal surgery and introduction of implants and devices, as well as to ventilator-associated pneumonia which increased during the COVID-19 pandemic. New preventive strategies and therapeutics for this single organism would be transformative for saving lives and improving quality of life.

**Figure 1:**
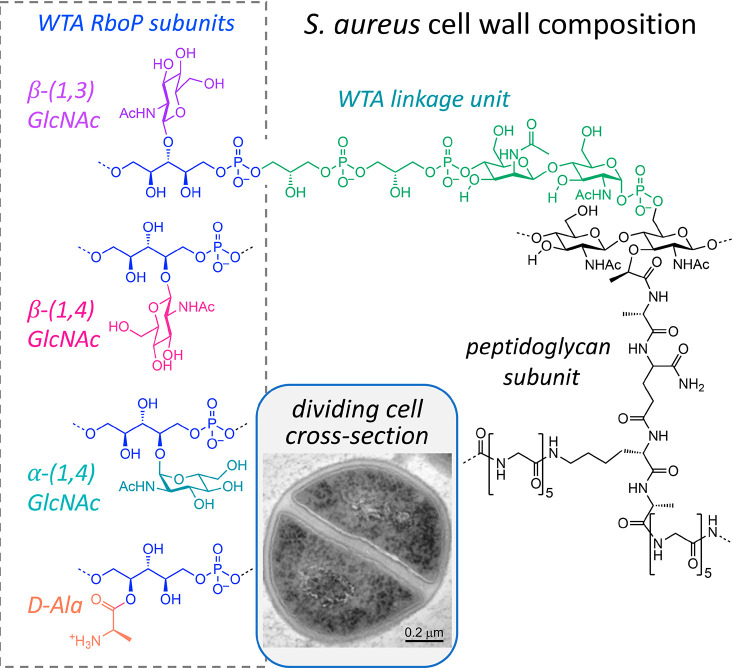
*Staphylococcus aureus* cell wall architecture and chemical structure. Cross-section visualization by transmission electron microscopy shows the thick cell wall surrounding the cell and through the septum in a dividing cell. The WTA backbone consists of up to approximately 40 ribitol-phosphate (RboP) units (with one unit shown in blue), that can be modified with α-GlcNAc, β-GlcNAc, and/or d-Ala. WTA is appended to MurNAc in PG via a linker (in green) containing a ManNAc-(β-1,4)-GlcNAc disaccharide and one to three glycerol-phosphate (GroP) units. Peptidoglycan contains a disaccharide and MurNAc-attached pentapeptide stem and glycyl bridge attached to the lysine side chain. The terminal bridge glycine is cross-linked to d-Ala of a neighboring stem through transpeptidation.

In this issue of *ACS Central Science*, Marchetti and co-workers shed new light on how two previously described monoclonal antibodies (mAb 4461 and mAb 4497) differentially recognize key glycopolymers termed teichoic acids produced by *S. aureus* that thread throughout and beyond the cell wall and present surface-exposed epitopes for antibody recognition.^2^ Their discoveries are relevant to vaccine development to prevent infection; to the design, evaluation, and development of mAb therapeutics to treat active infection or for prophylactic use, e.g., for surgery; and to the detection and diagnosis of *S. aureus* infection. *S. aureus* and other Gram-positive bacteria are defined by their thick cell wall that envelops and protects cells, wherein peptidoglycan and wall teichoic acid (WTA) partner in the assembly and remodeling of the mesh-like network (Figure 1). Peptidoglycan is considered the major architectural component, and WTAs are covalently attached to peptidoglycan. WTAs are important in bacterial cell division, influence colonization in healthy individuals (20–30% of humans are asymptomatically colonized), and contribute to pathogenesis in the host in ways that continue to be unraveled.^3^ The nearly universal production of teichoic acids by *S. aureus* strains renders them attractive candidates for the development of glycoconjugate vaccines or as targets for mAb recognition and therapy. Indeed, since the introduction of the first glycoconjugate vaccine in 1983 for *Haemophilus influenzae*, glycoconjugate vaccines against bacterial pathogens are now available to also prevent infections by *Streptococcus pneumoniae* and *Neisseria meningitidis*.^4^

**Figure 2:**
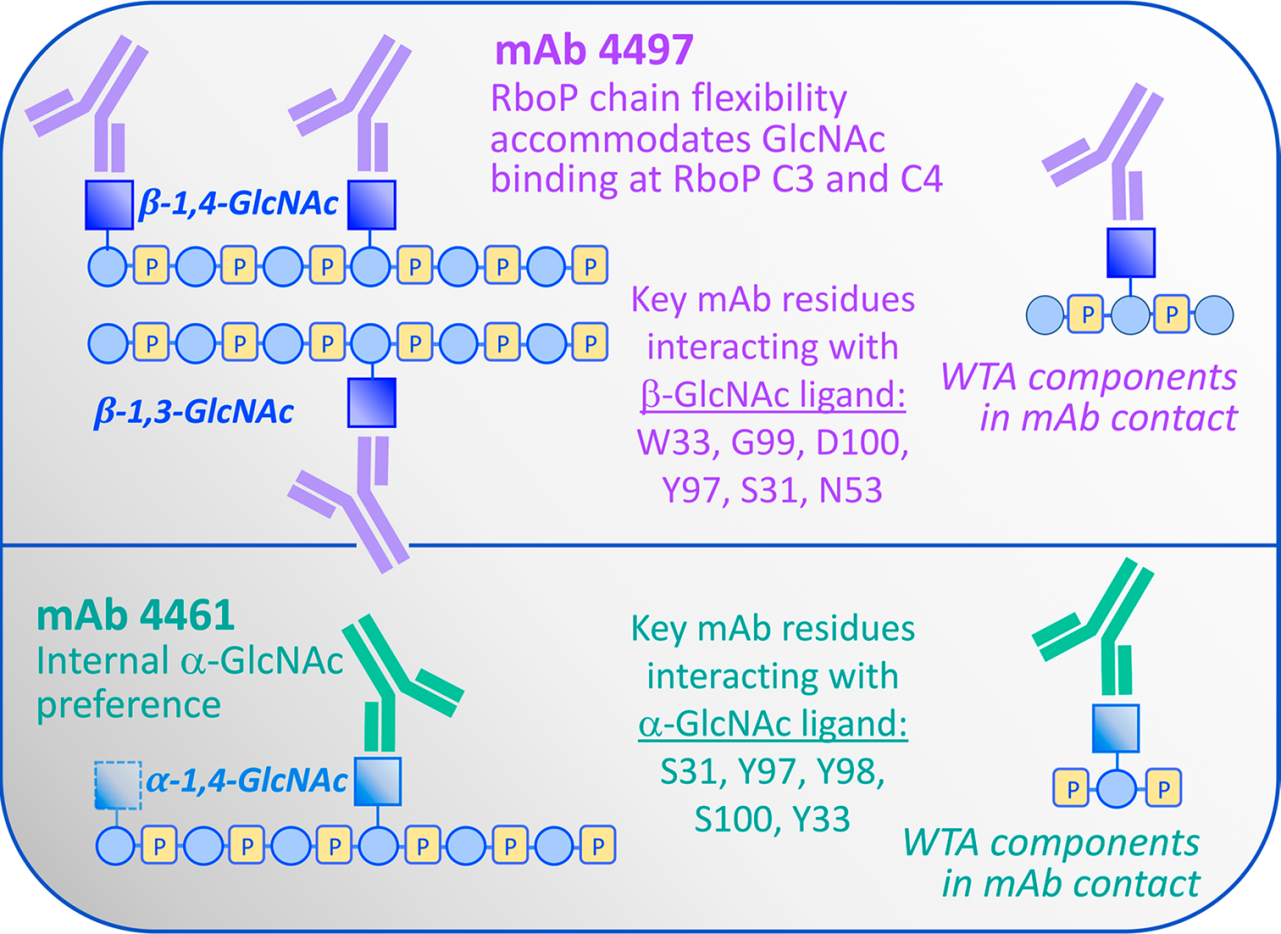
Summary of WTA-mAb binding specificity and interactions. Each mAb exhibits stereospecific GlcNAc recognition and exhibits differentiating molecular contacts with α-GlcNac versus the β-GlcNAc-appended WTA surrogates.

WTAs contain some structural variations in how their repeating units of ribitolphosphate (RboP) are modified, which influences immune detection and the host response. In *S. aureus*, *N*-acetylglucosamine (GlcNAc) can be attached to RboP to yield α-1,4-GlcNAc, β-1,3-GlcNac, and β-1,4-GlcNac modifications (Figure 1). WTA is also commonly d-alanylated through an ester linkage to RboP, presenting a positively charged amine that modulates the overall charge of the otherwise anionic RboP polymer (Figure 1). This new work leveraged specifically designed oligomeric synthetic constructs of WTA as previously reported by Codée, with one and also two GlcNAc modifications that are longer than many minimal epitopes used in other studies, to more fully understand the molecular basis of WTA binding to two mAbs: (1) mAb 4461 with binding specificity for α-GlcNAc WTA and (2) mAb 4497 with specificity for the β-GlcNAc WTAs. Earlier structure determinations preceded this work using minimal single ribose and phosphomonester ligands,^5,6^ while the authors here sought to investigate binding with more native-like WTA surrogates. Evaluation in their recently introduced teichoic acid microarray platform benchmarked the α and β binding specificities. New views of mAb binding to the WTA constructs emerged from an impressive combination of structure determinations of mAb-ligand complexes by X-ray crystallography, mapping of specific hydrogen bonds and atomic-level interactions between mAbs and WTA constructs by saturation-transfer difference (STD) NMR spectroscopy, and molecular dynamics simulations. The authors more fully uncovered the preference of mAb 4461 for recognizing internal α-1,4-GlcNAc residues. Furthermore, using this three-pronged structural approach, the authors observed that mAb 4497 exhibits key contacts not only to the flanking phosphates around β-GlcNAc-appended ribose (as was observed for mAb 4461 to α-GlcNAc WTA) but also to the next proximate ribose units, suggesting that dynamics in the WTA polymer backbone may underlie the ability of mAb 4497 to accommodate and bind β-GlcNAc attached to C3 or C4 ribose carbons (Figure 2). The structural analysis provides a framework for the intuitive concept that the strands of WTA running throughout the cell wall are flexible and that dynamics could relate to their function.

The focus on mAbs 4461 and 4497 harkens back to earlier pioneering work reported in 2015 and 2018, led by teams at Genentech, that generated monoclonal antibodies derived through B cell cloning of patient-derived antibodies that bind to WTA.^5,6^ The selection of mAbs was described as emerging through a broader antibody-antibiotic conjugate program to target and deliver antibiotic payloads. Such an approach could also be employed to deliver antibiotic-sparing virulence inhibitors such as inhibitors of biofilm matrix production or toxin secretion. Recent work by de Vor et al. (2022) additionally revealed that mAb 4497 recognizes *S. aureus* in biofilm communities and demonstrated detection of *S. aureus* in a subcutaneous implant mouse model.^7^

The potential impact to human health associated with new vaccine development and other clinical interventions for *S. aureus* infection would be tremendous. One can point to early studies to evaluate vaccine strategies and antibody therapies for *S. aureus* that achieved success in animal models but ultimately failed to advance in clinical trials in what are naturally referred to as setbacks. Yet, invaluable discoveries emerged from these failures with needed data, new lesson variations in how mice and people differ, and inspiration to chart out new paths of discovery. In addition to alternate glycoconjugate design opportunities, advances continue in introducing new adjuvants and immune-stimulatory strategies,^8^ including a recent approach that significantly extends the half-life of glyconjugates *in vivo*.^9^ New discoveries can emerge by returning to the earliest stages of the mAb 4491 program to identify and evaluate alternate patient-derived mAbs or to generate new ones based on newly designed synthetic WTA constructs. In the initial screening of mAbs reported by Lehar et al. (2015),^6^ patient-derived antibodies were evaluated for their binding capacity to cell wall preparations from bacteria extracted from the host. Performed above pH 7 and dependent on incubation times, cell wall preparations yield significant to complete hydrolysis and elimination of WTA d-Ala esters. Thus, antibodies that preferentially recognize native d-alanylated teichoic acids might have been missed. In addition, except for one study,^10^ it appears that most preparations of synthetic teichoic acids in *S. aureus* and other bacteria have lacked the inclusion of d-Ala or a related positively charged motif that could tune the charge and overall WTA epitope presented to antibodies. As Marchetti and co-workers described, it will be informative to examine interactions between mAb 4461 and mAb 4497 to d-alanylated or appropriately modified WTA surrogates and evaluate whether and how d-Ala conjugation might be accommodated in the binding pocket of mAbs.

Different challenges are faced in developing next-generation vaccines targeting *S. pneumoniae*, which like *S. aureus* is often found in asymptomatic carriage in healthy individuals. Current *S. pneumoniae* vaccines are designed to recognize up to 23 out of ∼100 surface carbohydrates that define serotypes. Yet, nonvaccine serotypes often increase in prevalence following vaccination and are competent for causing infection. Thus, hotly pursued avenues of development include expanding serotype coverage and identifying alternative strategies to target more universal surface structures. Juxtaposed to this race to keep up and expand antigen recognition to more *S. pneumoniae* serotypes, *S. aureus* strains all appear to harbor the common and possibly golden threads of teichoic acid that are so appealing to catch onto as a vaccine and therapeutic mAb target.
